# Modeling and study of the mechanism of dilated cardiomyopathy using induced pluripotent stem cells derived from individuals with Duchenne muscular dystrophy

**DOI:** 10.1242/dmm.019505

**Published:** 2015-05-01

**Authors:** Bo Lin, Yang Li, Lu Han, Aaron D. Kaplan, Ying Ao, Spandan Kalra, Glenna C. L. Bett, Randall L. Rasmusson, Chris Denning, Lei Yang

**Affiliations:** ^1^ Department of Developmental Biology, University of Pittsburgh School of Medicine, 530 45th Street, 8117 Rangos Research Center, Pittsburgh, PA 15201, USA; ^2^ Center for Cellular and Systems Electrophysiology, Departments of Physiology and Biophysics, SUNY, Buffalo, NY 14214, USA; ^3^ Department of Stem Cells, Tissue Engineering & Modelling (STEM), University of Nottingham, Nottingham, NG7 2RD, UK; ^4^ Departments of Obstetrics and Gynecology, and Physiology and Biophysics, SUNY, Buffalo, NY 14214, USA

**Keywords:** Dilated cardiomyopathy, Duchenne muscular dystrophy, Induced pluripotent stem cells

## Abstract

Duchenne muscular dystrophy (DMD) is caused by mutations in the dystrophin gene (*DMD*), and is characterized by progressive weakness in skeletal and cardiac muscles. Currently, dilated cardiomyopathy due to cardiac muscle loss is one of the major causes of lethality in late-stage DMD patients. To study the molecular mechanisms underlying dilated cardiomyopathy in DMD heart, we generated cardiomyocytes (CMs) from DMD and healthy control induced pluripotent stem cells (iPSCs). DMD iPSC-derived CMs (iPSC-CMs) displayed dystrophin deficiency, as well as the elevated levels of resting Ca^2+^, mitochondrial damage and cell apoptosis. Additionally, we found an activated mitochondria-mediated signaling network underlying the enhanced apoptosis in DMD iPSC-CMs. Furthermore, when we treated DMD iPSC-CMs with the membrane sealant Poloxamer 188, it significantly decreased the resting cytosolic Ca^2+^ level, repressed caspase-3 (CASP3) activation and consequently suppressed apoptosis in DMD iPSC-CMs. Taken together, using DMD patient-derived iPSC-CMs, we established an *in vitro* model that manifests the major phenotypes of dilated cardiomyopathy in DMD patients, and uncovered a potential new disease mechanism. Our model could be used for the mechanistic study of human muscular dystrophy, as well as future preclinical testing of novel therapeutic compounds for dilated cardiomyopathy in DMD patients.

## Abstract (precis)

**Highlighted Article:** Patient-derived induced pluripotent stem cells are used to establish an *in vitro* model of DMD-associated cardiomyopathy that could be used for future preclinical testing.

## INTRODUCTION

Duchenne muscular dystrophy (DMD) is the most common X-linked muscle-wasting disease. DMD occurs in ∼1 in 5000 male births (Mendell et al., 2012) and is caused by mutations in the *DMD* gene, which encodes dystrophin. The vast majority of DMD patients carry frame-shifting mutations in the *DMD* gene. Dystrophin connects the cytoskeleton to the extracellular matrix by interacting with a large protein complex, dystrophin glycoprotein complex (DGC). Dystrophin deficiency causes the loss of muscle membrane integrity and an increased susceptibility of muscle cells to stress-induced damages, which in turn leads to progressive weakness and wasting of skeletal and cardiac muscles. Dilated cardiomyopathy, which is due to heart muscle loss, together with increased fibrosis and cardiac arrhythmias, characterize DMD hearts (Eagle et al., 2002; Fayssoil et al., 2010; Romfh and McNally, 2010). It has been found that most DMD patients develop severe dilated cardiomyopathy in their early to middle teens and usually die of congestive heart failure in a few years from the onset of symptoms (Eagle et al., 2002; Fayssoil et al., 2010). Currently, cardiac complications, especially dilated cardiomyopathy, are the major lethal cause of late-stage DMD patients (Romfh and McNally, 2010). Thus, understanding the molecular mechanism of dilated cardiomyopathy is crucial for improving the survival of DMD patients.

Despite the progress in revealing the mechanism of skeletal muscle dystrophy, less attention has been directed to dilated cardiomyopathy in DMD patients. Currently, DMD has been studied with animal models in mouse, feline and canine (Ameen and Robson, 2010). The dystrophin-deficient C57Bl/10ScSn mdx (*mdx*) mouse is the most commonly used laboratory model of DMD. It has been reported that ventricular cardiomyocytes (CMs) of *mdx* mice exhibit some similar abnormalities to those found in DMD human heart cells (Quinlan et al., 2004), such as fragile muscle membrane and elevated resting cytosolic Ca^2+^. However, in contrast to DMD patients, *mdx* mice exhibit a much milder and much slower development of cardiac complications and have a normal life span (Quinlan et al., 2004). This suggests that different mechanisms underlie dilated cardiomyopathies in DMD patients versus *mdx* mice, which remains a major hurdle for studying the molecular etiology of human DMD cardiomyopathy, as well as conducting preclinical drug testing using DMD animal models. In addition, the availability of heart muscle biopsies from DMD patients is very limited, which prevents the mechanistic study and drug testing using native DMD patient heart cells and tissues. Recent advances in induced pluripotent stem cells (iPSCs) have circumvented this hurdle (Takahashi et al., 2007). iPSCs reprogrammed from patient-specific somatic cells carry the same genetic defects as original patients, and could be utilized to produce an unlimited number of patient-specific *de novo* CMs. Currently, single CMs have been derived from iPSCs of patients with various inherited heart diseases, including familial dilated cardiomyopathy (Sun et al., 2012), Leopard-syndrome-associated hypertrophic cardiomyopathy (Carvajal-Vergara et al., 2010), long QT Syndrome (Itzhaki et al., 2011) and familial hypertrophic cardiomyopathy (Han et al., 2014; Lan et al., 2013), to recapitulate disease phenotypes *in vitro*. However, iPS cells have not yet been used to uncover the molecular mechanisms of human inherited heart diseases.

**TRANSLATIONAL IMPACT**

**Clinical issue**

Duchenne Muscular Dystrophy (DMD) is the most common X-linked muscle-wasting disease. DMD is caused by mutations in the *DMD* gene, which encodes dystrophin. Dystrophin connects the cytoskeleton to the extracellular matrix by interacting with a large protein complex, the dystrophin glycoprotein complex (DGC). Dystrophin deficiency causes loss of muscle membrane integrity and an increased susceptibility of muscle cells to stress-induced damages, which in turn leads to progressive weakness and wasting of skeletal and cardiac muscles. Currently, dilated cardiomyopathy due to cardiac muscle loss represents one of the major lethal causes for individuals with late-stage DMD.

**Results**

Cardiomyocytes (CMs) were derived from DMD patient-specific induced pluripotent stem cells (iPSCs) and control iPSCs. DMD iPSC-CMs exhibited dystrophin deficiency, as well as increased levels of cytosolic Ca^2+^, mitochondria damage, caspase-3 (CASP3) activation and cell apoptosis. Additionally, by conducting whole transcriptional sequencing and translational analyses of high purity CMs derived from healthy or DMD iPSCs, a mitochondria-mediated signaling network [comprising the following cascade of molecular events: damaged mitochondria**→**DIABLO**→**XIAP**→**CASP3 cleavage**→**apoptosis] was found to account for the increased apoptosis in DMD iPSC-CMs. Furthermore, the membrane sealant Poloxamer 188 could prominently suppress cytosolic Ca^2+^ overload, repress CASP3 activation and decrease the amount of apoptosis in DMD iPSC-CMs.

**Implications and future directions**

In this study, DMD patient-derived iPSCs were utilized as an *in vitro* model to replicate the major phenotypes of dilated cardiomyopathy found in DMD-affected individuals, and to uncover the underlying disease mechanism. The study revealed a multi-staged pathway that is responsible for increased apoptosis in DMD CMs and that can be pharmacologically modulated. Thus, this *in vitro* system might also benefit the future preclinical testing of novel therapeutic compounds for dilated cardiomyopathy in DMD. 

In this study, we found that DMD patient-specific iPSC-derived CMs (iPSC-CMs) exhibited dystrophin deficiency, as well as increased levels of cytosolic Ca^2+^, mitochondria damage, CASP3 activation and cell apoptosis. Additionally, by conducting whole transcriptional sequencing and translational analyses of high-purity CMs derived from healthy and DMD iPSCs, we found the increased apoptosis of DMD iPSC-CMs could be triggered by a mitochondria-mediated signaling network from damaged mitochondria→DIABLO→XIAP→CASP3 cleavage→apoptosis. Furthermore, we observed that the membrane sealant Poloxamer 188 could prominently suppress cytosolic Ca^2+^ overload, repress CASP3 activation and decrease the proportion of cleaved caspase-3 (C-CASP3)-positive apoptotic CMs in DMD iPSC-CMs. Overall, using DMD patient-derived iPSCs, this study establishes a novel *in vitro* model for recapitulating disease phenotypes, studying molecular disease mechanism and future preclinical testing of novel therapeutic compounds for dilated cardiomyopathy in DMD patients.

## RESULTS

### Characterizations of DMD patient-specific iPSCs

**Fig. 1.**
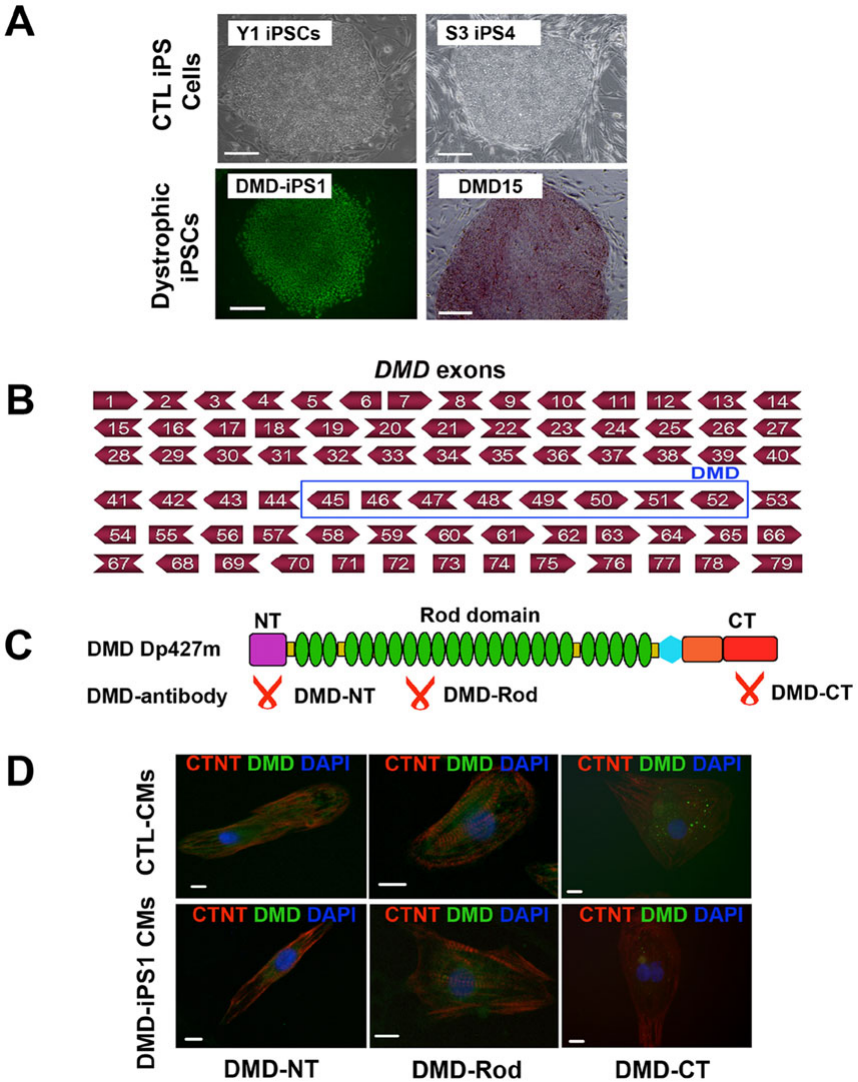
**CM differentiation from control and DMD iPSCs.** (A) Representative images showing morphologies of control Y1, S3 iPS4 and DMD iPSCs. DMD-iPSC1 cells were positive for OCT4 immunostaining (green) and DMD15 iPSCs were positive for live staining of pluripotency surface marker, alkaline phosphatase (red). Scale bars: 100 μm. (B) Schematic showing dystrophin mutations in DMD-iPS1 and DMD15 iPSCs. Both DMD-iPS1 and DMD15 iPSCs have the same DMD mutation. (C) Schematic showing the Dp427m DMD isoform expressed in iPSC-CMs and the locations where anti-DMD antibodies bind to the N-terminal (NT), Rod domain and C-terminal (CT) of dystrophin. (D) Immunostaining of dystrophin (green) with the anti-DMD antibodies described in C in control S3 and DMD-iPS1-cell-derived CMs (CTNT, red). Scale bars: 10 µm.

In this study, we obtained two previously established DMD iPSC lines, DMD-iPS1 (Park et al., 2008) and DMD15 (Dick et al., 2013) (Fig. 1A). Additionally, two established normal pluripotent stem cell lines were utilized as the healthy control, which are Y1 iPSCs and S3 iPS4 cells. Human Y1 iPSCs (Lin et al., 2012) were previously generated from human dermal fibroblasts (HDF-α; Cellapplications, San Diego, CA), and S3 iPSCs (Carvajal-Vergara et al., 2010) were previously generated from fibroblasts of a healthy donor. Similar to in previous reports (Park et al., 2008; Takahashi et al., 2007), the expression of retroviral transgenes encoding OCT4, SOX2, KLF4 and c-MYC were uniformly diminished in all iPSC lines (supplementary material Fig. S1a).

Next, we confirmed the *DMD* mutations in DMD iPSCs using normal PCR. We identified an out-of-frame deletion in *DMD* exons 45-52 in both DMD-iPS1 and DMD15 iPSCs (Fig. 1B, supplementary material Fig. S1b). No *DMD* mutations were found in the healthy control iPSCs. Given that over a thousand types of *DMD* mutations have been observed in DMD patients (Flanigan et al., 2009), here we only used DMD-iPS1 and DMD15 iPSCs to represent a specific group of DMD patients who carry the same type (gene deletion) of site-specific *DMD* deletion.

### Cardiomyocyte differentiation from human iPSCs

**Fig. 2.**
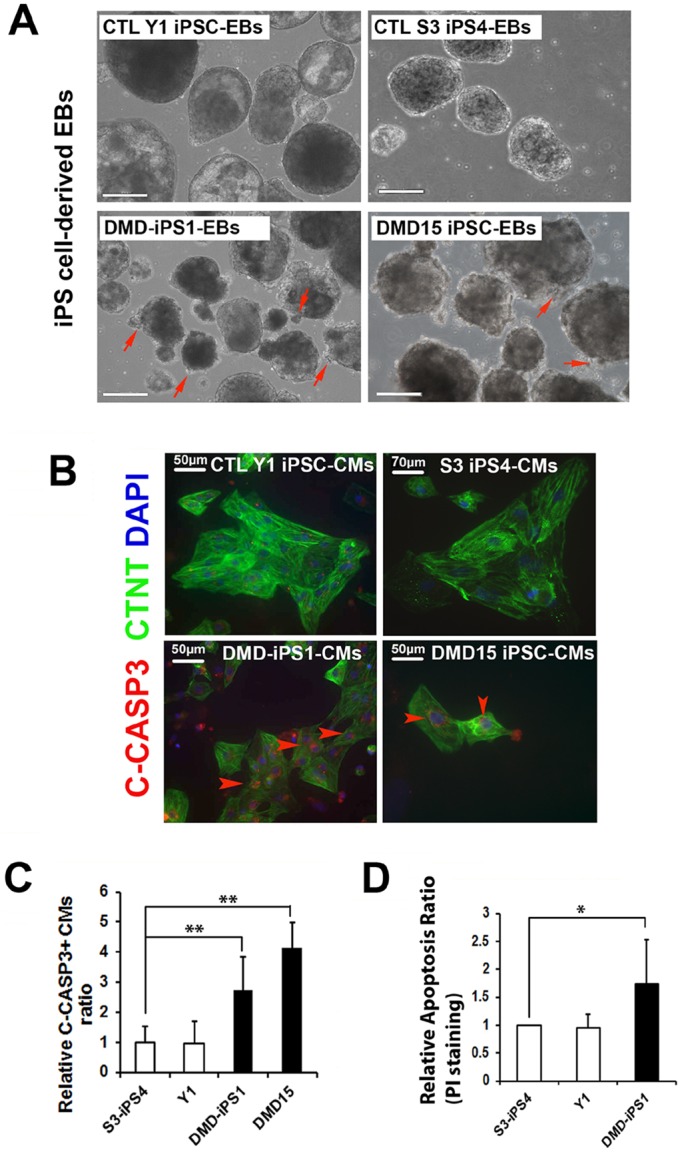
**Detection of apoptosis.** (A) Representative images showing iPSC-EBs at day 22 of differentiation. Red arrows indicate the cell debris on iPSC-EBs. Scale bars: 200 μm. (B) Representative immunostaining images of cleaved caspase-3 (C-CASP3, red) in iPSC-CMs (CTNT, green). Red arrowheads indicate the C-CASP3+ CMs. Scale bars: 50 µm. (C) Quantification of C-CASP3-positive CMs in control and DMD iPSC-CMs (Y1, *n*=657; S3, *n*=1129; DMD-iPS1, *n*=860; DMD15, *n*=53). (D) CMs with PI staining followed by FACS quantification of DNA fragmentation. All results are mean±s.d. of four independent experiments. **P<0.01, *P<0.05 (two-tailed Student's *t*-test).

Embryoid bodies (EBs) were formed from the control and DMD iPSCs (Fig. 2A) to induce cardiomyocyte differentiation using our previously established method (Lin et al., 2012) (supplementary material Fig. S2a,b). This cardiac differentiation method was developed from our previous cardiovascular differentiation protocol in human embryonic stem cells (ESCs) (Yang et al., 2008) and has been utilized for modeling Leopard-syndrome-associated hypertrophic cardiomyopathy (Carvajal-Vergara et al., 2010), dilated cardiomyopathy (Sun et al., 2012) and familiar hypertrophic cardiomyopathy (Lan et al., 2013) with patient-specific iPSCs. At day 22 of differentiation, iPSC-derived EBs exhibited spontaneous contractions (supplementary material Movies 1-3). Next, we conducted RT-PCR to detect the expressions of *DMD* isoforms in undifferentiated iPSCs and iPSC-CMs (supplementary material Fig. S2c). The constitutively expressed short *DMD* isoform Dp71 was found in undifferentiated iPSCs, and the muscle-specific long Dp427m isoform was solely detected in iPSC-CMs. The intermediate Dp140 isoform was not detectable in either iPSCs or iPSC-CMs. This indicates that the deficiency of functional Dp427m isoform is the major cause of dilated cardiomyopathy in DMD patients, which is consistent with previous studies from DMD animal models (Ameen and Robson, 2010; Quinlan et al., 2004). Furthermore, three anti-human dystrophin antibodies (Leica), which recognize the N-terminus (NT), Rod domain (Rod) or C-terminus (CT) of dystrophin, were utilized to detect the dystrophin protein levels in control and DMD iPSC-CMs (Fig. 1C), respectively. As shown in Fig. 1D, iPSC-CMs were recognized with an anti-cardiac troponin T (CTNT) antibody and dystrophin was detected by all three anti-dystrophin antibodies in control iPSC-CMs. A decreased level of dystrophin was detected in DMD iPSC-CMs when compared with that in control iPSC-CMs, which is consistent with the western blot results using the same antibodies (see Fig. 4C, left panel).

### Increased cell death in DMD iPSC-CMs

Dystrophic heart is characterized by dilated cardiomyopathy, which is caused by a progressive CM loss (Fayssoil et al., 2010; Mendell et al., 2012; Yilmaz et al., 2008). Therefore, we sought to examine cell apoptosis in DMD iPSC-CMs. Interestingly, the outside surfaces of EBs from both control Y1 and S3 iPSCs were consistently very smooth, indicating a healthy growth and differentiation status (Fig. 2A). However, a significantly increased amount of cell debris was found on the surfaces of DMD iPSC-derived EBs (iPSC-EBs), implying an enhanced cell death in DMD iPSC-EBs (Fig. 2A; supplementary material Movies 1-3). Next, we dissociated iPSC-EBs and stained CMs with the anti-C-CASP3 antibody. C-CASP3 is the predominant factor in the execution-phase of cell apoptosis (Porter and Janicke, 1999). An increased proportion of CTNT and C-CASP3 double-positive cells was found in DMD iPSC-CMs (Fig. 2B,C) when compared with control CMs, indicating that there is enhanced cell apoptosis in DMD iPSC-CMs. Furthermore, we utilized propidium iodide (PI) staining followed by fluorescence-activated cell sorting (FACS) analysis to quantify DNA fragmentation in control and DMD iPSC-CMs, which has been previously utilized for measuring cell apoptosis (Riccardi and Nicoletti, 2006). Here, we only compared DNA fragmentations among iPSCs with similar CM generation efficiencies (supplementary material Fig. S2b). As shown in Fig. 2D, increased levels of DNA fragmentation were observed in DMD-iPS1 iPSC-CMs when compared with the control Y1 and S3 iPSC-CMs. All these results demonstrate an increased level of apoptosis in DMD iPSC-CMs compared with control iPSC-CMs. However, as previously reported, the cleavage of un-cleaved CASP3 (called pro-CASP3) into active C-CASP3 could be activated through multiple pathways (Porter and Janicke, 1999). Uncovering the upstream signals that activate CASP3 cleavage in DMD iPSC-CMs is crucial for revealing the molecular mechanism underlying DMD-associated dilated cardiomyopathy.

### Whole-transcriptome sequencing of iPSC-CMs

**Fig. 3.**
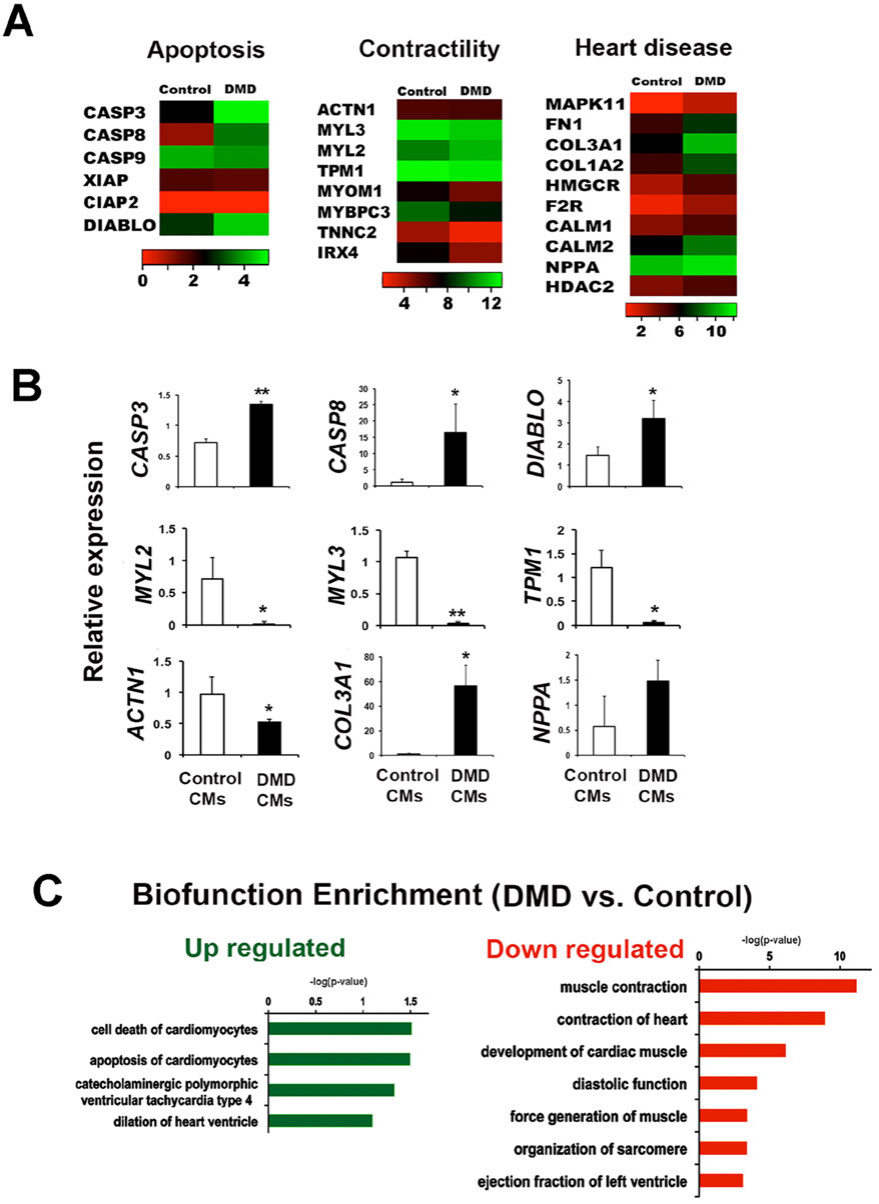
**Whole-transcriptome sequencing of DMD iPSC-CMs.** (A) Whole transcriptome sequencing of iPSC-EBs at day 22 of differentiation. Fragment per kilobase of exon per million reads (FPKM) of genes from DMD iPS1 CMs (*n*=3 each) and control S3 iPSC-CMs (*n*=3) were averaged and compared. The FPKM value indicates the relative expression level of a sequenced gene. Heat maps were drawn with log2 of FPKM (mean±s.d. of triplicate experiments) for representative genes in control versus DMD CMs. (B) Validation of gene expression by q-RT-PCR. Error bars show s.d. of triplicate experiments. *P<0.05, **P<0.01 (two-tailed Student's *t*-test). (C) IPA analysis of the differentially expressed genes in DMD-iPS1 CMs versus control S3 iPSC-CMs. Green indicates the upregulated, and red indicates the downregulated bio-function categories in DMD iPSC-CMs when compared with control iPSC-CMs.

In order to uncover the molecular mechanism underlying enhanced cell apoptosis in DMD iPSC-CMs, first we conducted whole-transcriptome sequencing to detect the whole transcriptional changes between DMD iPSC-CMs and control iPSC-CMs. Here, we chose S3 control and DMD-iPS1-cell-derived CMs for sequencing analysis because these cell lines consistently gave rise to over 75% CMs at day 22 of differentiation using our established protocol (supplementary material Fig. S2b), whereas CM formation efficiency from DMD15 iPSCs using the same protocol was only ∼26%. It is important to note that we directly sequenced iPSC-derived beating EBs, rather than iPSC-CMs enriched using previously reported FACS method (Lin et al., 2012), because we found most of the apoptotic iPSC-CMs were broken into pieces by the high hydrodynamic stress from FACS sorter and the loss of pro-apoptotic or apoptotic iPSC-CMs would significantly affect the transcriptional profiles of DMD iPSC-CMs. By comparing the transcriptional profiles of control S3 iPSC-CMs versus DMD-iPS1-CMs, we found that approximately 1200 genes exhibited significant expression changes (*P*<0.05, Student's *t*-test). Interestingly, we found that a number of genes that play an essential role in regulating apoptosis, such as *CASP3*, *CASP8*, *CASP9* and *XIAP*, genes controlling CM contractility, such as *MYL2*, *MYL3*, α-actinin (*ACTN1*) and α-tropomyocin (*TPM1*), and genes involved in heart diseases, such as *MAPK11*, *COL3A1* and *CALM1*, were abnormally expressed in DMD-iPS1 CMs when compared with control S3 iPSC-CMs (Fig. 3A). Quantitative real-time PCR (q-RT-PCR) validated the sequencing results, as shown in Fig. 3B. Finally, bio-functional enrichment analysis of the genes that showed the same expression changes (i.e. either upregulated or downregulated) in DMD iPSC-CMs versus control iPSC-CMs was performed using IPA (Ingenuity Pathway Analysis Software). Interestingly, bio-functional categories related to heart disease conditions, such as ‘Cell death of CMs’, ‘Dilation of heart ventricle’ and ‘Ventricular tahcycardia’, were positively enriched, whereas functions related to ‘Muscle development and contractility’ were negatively enriched in DMD iPSC-CMs when compared with control iPSC-CMs (Fig. 3C). These enrichment categories are highly consistent with observed cardiac abnormalities within DMD patients (Fayssoil et al., 2010; Finsterer and Stollberger, 2008; Romfh and McNally, 2010; Yilmaz et al., 2008), indicating the possibility to uncover molecular mechanisms of DMD-associated heart disease using DMD iPSC-CMs.

### A mitochondrial-mediated signaling network underlying enhanced CM death in DMD iPSC-CMs

**Fig. 4.**
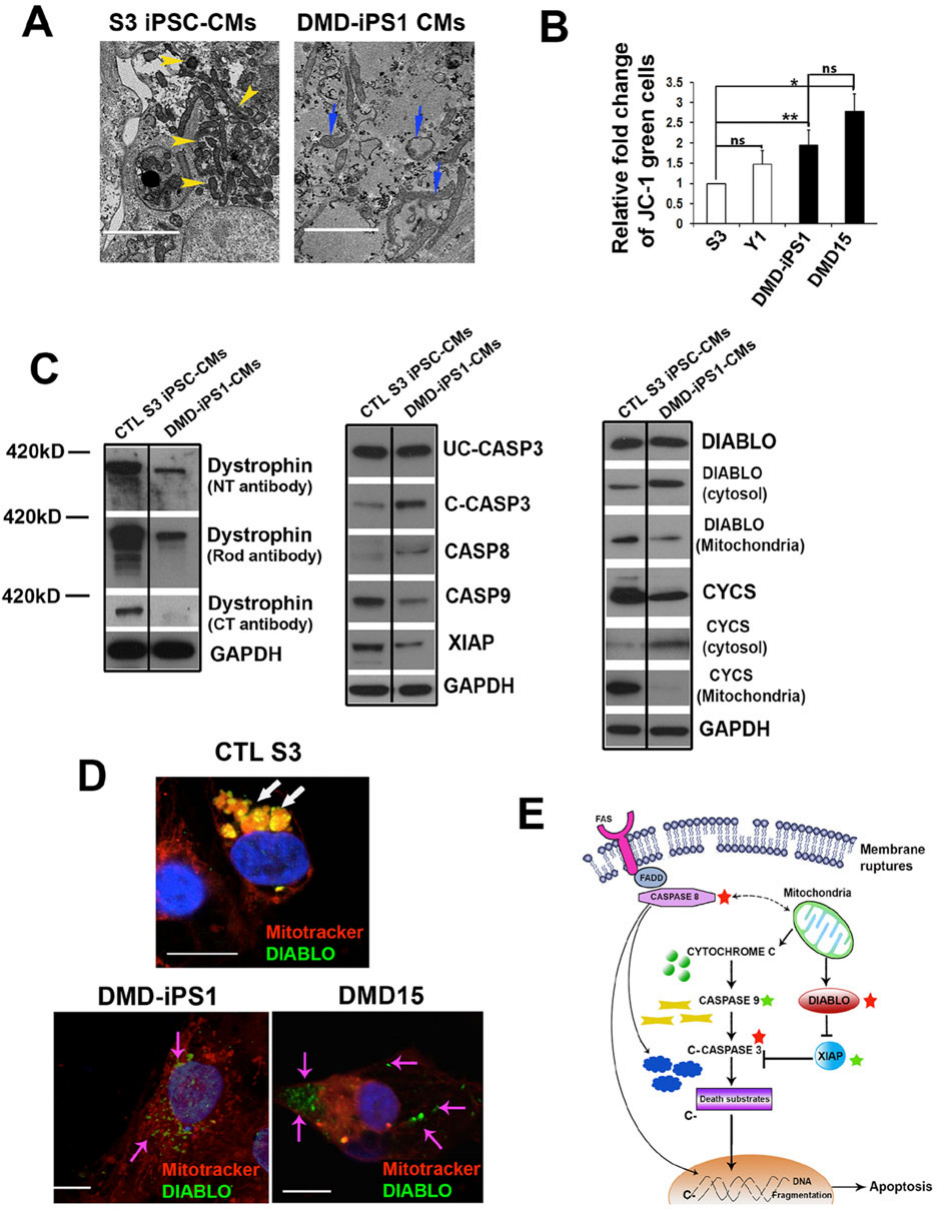
**Mitochondria-mediated apoptosis network in DMD iPSC-CMs.** (A) TEM of mitochondria in iPSC-CMs. Yellow arrowheads indicate the healthy mitochondria and blue arrows indicate the swollen mitochondria. Scale bars: 2 μm. (B) Green JC-1 staining indicates damaged mitochondria with disrupted membrane potential. Quantification of green JC-1 was conducted in all day 22 control and DMD iPSC-CMs. The *y*-axis indicates the relative change of the ratio of cells showing JC-1 green. Error bars show s.d. of five independent experiments. *P<0.05, **P<0.01 (two tailed Student's *t*-test). (C) Western blot analysis of day 22 control and DMD iPSC-EBs (left and middle panels). Mitochondria-free cytosol and mitochondria were fractionated from iPSC-EBs, followed by western blotting to detect the expressions of DIABLO and CYCS (right panel). The same amounts of samples were loaded based on their GAPDH expression levels before fractioning. UC-CASP3, uncleaved CASP3. (D) iPSC-CMs were stained with MitoTracker dye (red) to stain mitochondria and anti-DIABLO antibody (green). White arrows indicate DIABLO in mitochondria. Pink arrows indicate the cytosolic DIABLO released from mitochondria. Scale bars: 10 μm. (E) A schematic depicting the mitochondria-mediated apoptosis network in DMD iPSC-CMs. Red stars indicate the increased, and green stars indicate the decreased protein levels in DMD iPSC-CMs compared with control iPSC-CMs.

The whole-transcriptome sequencing analysis uncovered enhanced expression of *CASP3* and *DIABLO*, whereas *XIAP* was decreased in DMD iPSC-CMs (Fig. 3A,B), implying a possible role of mitochondria (Verhagen et al., 2000) in mediating apoptosis of DMD iPSC-CMs. However, such gene transcriptional changes might not reflect the changes of those genes at the protein level. Thus, we next sought to study the mechanism of enhanced apoptosis in DMD iPSC-CMs at the translational level. As the first step, we conducted transmission electron microscopy (TEM) to examine mitochondria morphology. Compared to the compact mitochondria observed in control iPSC-CMs, disrupted and swollen mitochondria were observed in DMD iPSC-CMs (Fig. 4A). Because mitochondrial disruption could be indicated by changes of membrane potential, control and DMD iPSC-CMs were stained with a MitoProbe™ JC-1 dye, which exhibits potential-dependent accumulation in mitochondria, for measuring mitochondrial health (Smiley et al., 1991). FACS analysis of iPSC-CMs post JC-1 staining revealed an increased ratio of damaged mitochondria in DMD iPSC-CMs than in control iPSC-CMs (Fig. 4B; supplementary material Fig. S3).

Second, we performed western blotting to compare the expression levels of several key components within the mitochondria-mediated apoptosis pathway in day 22 EBs derived from control S3 and DMD-iPS1 cells (Fig. 4C), followed by quantification analysis (supplementary material Fig. S5). Western blot analysis (Fig. 4C, left panel) revealed altered-size (truncated) immunoreactive dystrophin bands in DMD iPSC-CMs compared with control iPSC-CMs, when immunostained with antibodies against the N-terminus and Rod region of dystrophin. When immunostained with the antibody specific for the C-terminus of dystrophin, no dystrophin band was detected in the DMD1-iPS1 CMs. This is consistent with the detected out-of-frame *DMD* deletion within the Rod region in DMD-iPS1 cells (Fig. 1B). Quantitative studies demonstrated a decreased abundance of truncated dystrophins in DMD iPSC-CMs when compared with control iPSC-CMs (supplementary material Fig. S4). Although no significant expression change of un-cleaved CASP3 (UC-CASP3) was found between control and DMD iPSC-CMs, enhanced levels of C-CASP3 were observed in DMD iPSC-CMs, indicating the existence of an activated upstream cascade to catalyze CASP3 cleavage (Fig. 4C, middle panel). It has been previously revealed that XIAP could inhibit CASP3 activation (Holcik et al., 2001; Porter and Janicke, 1999; Verhagen et al., 2000). Interestingly, the expression level of XIAP (Holcik et al., 2001) in DMD iPSC-CMs was significantly lower than that in control iPSC-CMs. Given that XIAP is a direct target of DIABLO (which is also referred to as second mitochondria-derived activator of caspases, SMAC) (Verhagen et al., 2000), we next sought to examine the expression level of DIABLO in control and DMD iPSC-CMs. Because DIABLO inhibits XIAP only when it is released from mitochondria to enter the cytosol (Verhagen et al., 2000), we fractionated iPSC-CMs to obtain both mitochondrial and mitochondria-free cytosolic fractions, followed by western blotting to detect DIABLO in each fraction (Fig. 4C, right panel). A significant increase of DIABLO was detected in the mitochondria-free cytosolic fraction, and a prominent decrease of DIABLO was detected in the mitochondrial fraction of DMD iPSC-CMs when compared with control iPSC-CMs, indicating the cytosolic release of DAIBLO from the damaged mitochondria in DMD iPSC-CMs. Similarly, a cytosolic release of cytochrome *c* (CYCS) (Kroemer et al., 1998) from damaged mitochondria was also observed in DMD iPSC-CMs (Fig. 4C, right panel). Previous reports have demonstrated that CYCS could activate CASP3 through activating CASP9 (Jiang and Wang, 2000; Kroemer et al., 1998). Interestingly, a downregulated expression level of CASP9 was observed in DMD iPSC-CMs when compared with control iPSC-CMs (Fig. 4C, middle panel; supplementary material Fig. S4), indicating that the cleavage of UC-CASP3 in DMD iPSC-CMs could be possibly mediated by other caspase(s).

Finally, co-staining of iPSC-CMs with mitochondria dye MitoTracker (red) and DIABLO (green) revealed the retention of DIABLO in mitochondria from control iPSC-CMs, whereas the release of DIABLO from mitochondria into cytosol was observed in DMD iPSC-CMs (Fig. 4D, supplementary material Fig. S5). Taken together, all these data reveal that a common mitochondria-mediated signaling network underlies enhanced apoptosis in DMD iPSC-CMs (Fig. 4E), which could account for dilated cardiomyopathy in DMD patients with a Rod-region-specific dystrophin gene deletion.

### Ca^2+^ handling in DMD iPSC-CMs

**Fig. 5.**
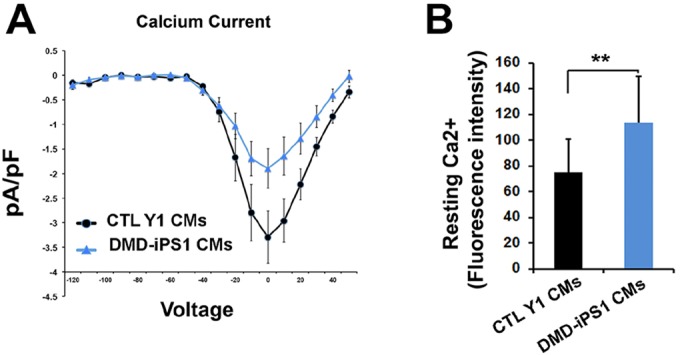
**Ca^2+^ handling of control and DMD iPSC-CMs.** (A) Measurement of L-type Ca^2+^ currents in single iPSC-CMs using patch clamp. (Control, *n*=12; DMD-iPS1, *n*=12). (B) Quantification of resting [Ca^2+^]_i_. (Control, *n*=80; DMD-iPS1, *n*=62.) All error bars show s.d. **P<0.01 (two-tailed Student's *t*-test).

Previous studies have observed abnormal Ca^2+^ handling in CMs from *mdx* mice (Williams and Allen, 2007; Yasuda et al., 2005), and it has been reported that the sustained elevation in Ca^2+^ levels through L-type Ca^2+^ channels precedes cytochrome *c* release from the mitochondria during cell apoptosis (Boehning et al., 2003; January and Riddle, 1989). Therefore, we measured the L-type Ca^2+^ current of single iPSC-CMs using whole-cell patch clamp. Compared with control iPSC-CMs, a profound reduction of the L-type Ca^2+^ current was found in DMD iPS1 CMs (Fig. 5A), consistent with previous observations from *mdx* mice (Koenig et al., 2012). Next, we examined the resting cytosolic Ca^2+^ concentration ([Ca^2+^]_i_) of iPSC-CMs using Ca^2+^ imaging. An elevated level of [Ca^2+^]_i_ was found in DMD iPSC-CMs compared with control iPSC-CMs (Fig. 5B). These results reveal that there is an altered Ca^2+^ homeostasis in human DMD iPSC-CMs, which might function as the upstream factor to trigger the apoptosis of CMs in DMD heart.

### P188 suppresses apoptosis of DMD iPSC-CMs

**Fig. 6.**
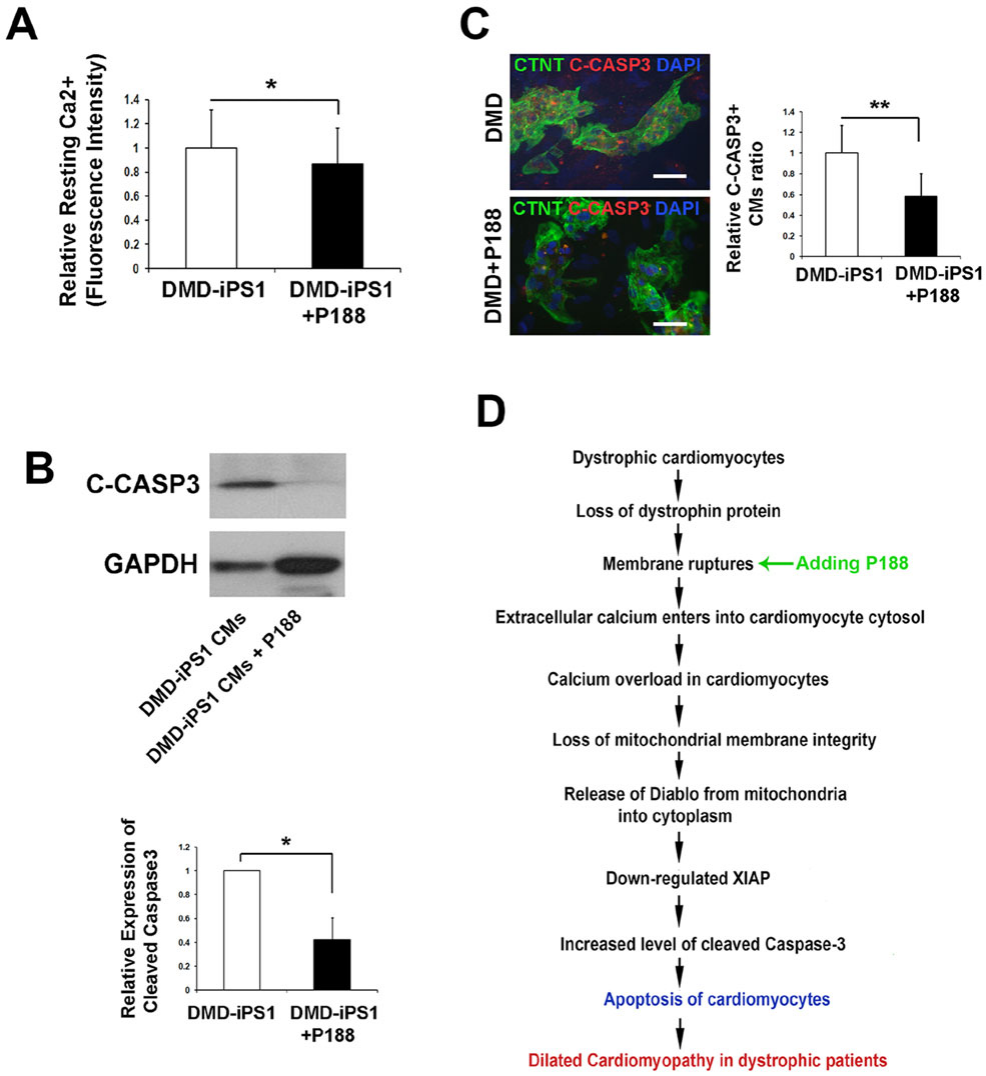
**P188 suppresses CASP3 cleavage in DMD iPSC-CMs.** (A) DMD iPSC-CMs were treated with P188 (1 mg/ml) for 7 days, followed by Ca^2+^ imaging. P188 suppressed the resting [Ca^2+^]_i_ (DMD-iPS1 CMs, *n*=62; DMD-iPS1 CMs+P188, *n*=36). *P<0.05. (B) P188 suppressed cleavage of CASP3 in DMD iPSC-CMs. Western blot analysis of DMD iPSC-CMs with and without P188 treatment (1 mg/ml) for 7 days. Representative images of western blots are shown in the upper panel and statistical analysis of band intensity is shown in the lower panel. *P<0.05 (*n*=3). (C) Representative images of C-CASP3 immunostaining (left panel) and quantification of C-CASP3+ CTNT+ CM ratios (right panel) in DMD-iPS1 CMs with and without P188 treatment (DMD-iPS1 CMs, *n*=546; DMD-iPS1 CMs+P188, *n*=332). **P<0.01. Scale bars: 10 µm. (D) A schematic summary of the possible mechanism, as well as potential therapeutic strategies for dilated cardiomyopathy in DMD patients. In all graphs, error bars show the s.d., and the *P* values were calculated with a two-tailed Student's *t*-test.

We next tested whether suppressing cytosolic Ca^2+^ overload could be an effective strategy for repressing apoptosis in DMD iPSC-CMs. The membrane sealant Poloxamer P188 has been previously found to maintain the membrane integrity and decrease stretch-induced Ca^2+^ overload in isolated CMs from *mdx* mice (Yasuda et al., 2005). We found P188 (1 mg/ml) treatment for 7 days (day 18-25 of differentiation) significantly decreased [Ca^2+^]_i_ of DMD iPSC-CMs (Fig. 6A). Interestingly, we observed that P188 treatment significantly repressed CASP3 activation in DMD-iPS1 CMs (Fig. 6B), and consequently suppressed the ratios of C-CASP3+ apoptotic CMs in DMD-iPS1 CMs (Fig. 6C). Furthermore, we found that P188 treatment significantly repressed the expression of DIABLO, but not XIAP in DMD iPSC-CMs (supplementary material Fig. S6). These data indicate that P188 might suppress CASP3 activation by decreasing cytosolic Ca^2+^ overload in DMD CMs and demonstrate that improving cell membrane integrity could be an effective strategy for attenuating and/or preventing CM loss in DMD patient hearts. Taken together, our results suggest that apoptosis of DMD iPSC-CMs is mainly triggered through DIABLO, XIAP and CASP3, rather than through cytochrome *c* and a CASP9 cascade (Fig. 6D).

In conclusion, using DMD patient-specific iPSC-CMs, we recapitulated the major cardiac abnormalities of dilated cardiomyopathy, uncovered a potential new molecular mechanism and explored the potential therapeutic strategies of dilated cardiomyopathy in DMD patients. This study might have broad implications for understanding cardiac and skeletal muscle failures in DMD patients, as well as for testing novel therapeutic compounds using a patient specific iPSC model.

## DISCUSSION

The human *DMD* gene, which encodes dystrophin, contains 79 exons and approximately 2.6 million base pairs (bp) of genomic DNA. In muscle cells, dystrophin binds to the cytoskeleton by associating with actin filaments at its N-terminus, and the C-terminus of dystrophin interacts with dystroglycan to link with extracellular matrix. This DGC stabilizes the sarcolemma and mediates signals between the cytoskeleton, membrane and extracellular matrix. Over 1000 mutations in the *DMD* gene have been identified as being responsible for DMD (Flanigan et al., 2009). The lack of functional dystrophin typically causes the instability of myofibril plasma membrane, which in turn leads to progressive damage and loss of skeletal and cardiac muscles. However, it has been found that DMD patients show variable disease penetrance and phenotypic expressivities. All these indicate that, beyond the *DMD* mutations, the patient-specific genetic background could significantly impact on the severities of clinical symptoms. Additionally, given the genetic heterogeneity of DMD patients, the limited laboratory animal models might not represent the >1000 mutations in DMD patients. Thus, mechanistic studies, as well as the preclinical testing of therapeutics for DMD patient associated dilated cardiomyopathy, require a patient-specific and disease-specific laboratory model.

This study sought to establish a novel *in vitro* model for those purposes. We induced CMs from both control and DMD iPSCs with high purities, and then performed mechanistic studies at the whole transcriptional level using whole-transcriptome sequencing, and at the protein translational level using western blot and immunostaining assays. In this study, the DMD iPSC-CM model manifests cardiac abnormalities that are also seen in DMD patients, such as dystrophin deficiency, resting Ca^2+^ overload and increased apoptosis. Most importantly, by conducting whole-transcriptome sequencing and IPA bio-functional enrichment analysis, we found that the differently expressed genes in DMD iPSC-CMs versus control iPSC-CMs were enriched into bio-functional categories that are highly consistent with the cardiac abnormalities observed in DMD patient hearts (Fayssoil et al., 2010; Finsterer and Stollberger, 2008; Romfh and McNally, 2010; Yilmaz et al., 2008), such as increased CM death and decreased cardiac functionality. Therefore, our study using DMD patient-specific iPSC-CMs our results prove the feasibility of modeling and have uncovered a molecular mechanism of dilated cardiomyopathy.

In this report, our mechanistic studies have revealed that a mitochondria-mediated network underlies the apoptosis of DMD iPSC-CMs. Findings from this mechanistic study might contribute to the identification of novel therapeutic targets for dilated cardiomyopathy in DMD patients. For example, we observed the endogenous level of XIAP, which is an apoptosis inhibitor that acts to suppress the transduction of pro-apoptosis signal from cytosolic DIABLO to activate CASP3, was significantly downregulated in DMD iPSC-CMs compared with the control iPSC-CMs. This suggests that XIAP is a potential therapeutic target of dilated cardiomyopathy in DMD patients. Additionally, we observed dystrophin-deficiency induced Ca^2+^ overload in DMD iPSC-CMs, suggesting that targeting the upstream of this mitochondria-induced apoptosis network (Fig. 6D) might be more efficient for repressing apoptosis of DMD iPSC-CMs. Based on these findings, we then tested whether a membrane sealant P188 could effectively repress cell apoptosis in DMD iPSC-CMs. P188 has been reported to decrease stretch-induced Ca^2+^ overload in isolated CMs from *mdx* mice by maintaining the membrane integrity (Yasuda et al., 2005). The therapeutic role of P188 in dilated cardiomyopathy of human DMD patients remains unclear. We found P188 treatment significantly decreased apoptosis of DMD iPSC-CMs by suppressing the activation of CASP3. Unfortunately, due to the concern over its toxicity, P188 might not be suitable for long-term use in humans (Moloughney and Weisleder, 2012). However, these studies have indicated that developing new therapeutic strategies to target the apoptosis initiation stage could efficiently prevent dilated cardiomyopathy. Additionally, our results demonstrate that DMD iPSC-CMs could serve as a new *in vitro* assay system for preclinical testing of novel therapeutic compounds.

It is important to note that all results of this study only represent findings from a specific group of DMD patients who carry the same type of dystrophin mutation (gene deletion) within the same Rod region. Given that dystrophin deficiency is the major cause of cell death in skeletal and heart muscles (Eagle et al., 2002; Fayssoil et al., 2010; Finsterer and Stollberger, 2008; Mendell et al., 2012; Romfh and McNally, 2010), we expect our findings would benefit the mechanistic studies of CM loss in DMD patients with other types of DMD mutations, as well as skeletal muscle loss in DMD patients. Additionally, the whole working strategy established in this study could be utilized for studying molecular mechanisms of other human inherited heart diseases, such as inherited hypertrophic cardiomyopathy (Han et al., 2014).

## MATERIALS AND METHODS

### Human iPSC culture and differentiation

Two clones of DMD-iPS1 cells (23D1 and 23D2) were previously generated from a 6-year-old DMD male patient and characterized by Park et al. (2008). DMD15 iPSCs were previously established from a 13-year-old DMD male patient and fully characterized (Dick et al., 2013). Healthy control human Y1 iPS cells were generated from human dermal fibroblasts (HDF-α; Cellapplications, San Diego, CA) in our laboratory and S3 iPS4 cells were generated from fibroblasts of a healthy control donor as previously described (Carvajal-Vergara et al., 2010; Lin et al., 2012). Human iPSCs were maintained on mitotically inactivated mouse embryonic fibroblasts (MEFs) in human ESC medium containing DMEM/F12 (Invitrogen), 20% (vol/vol) KSR (Invitrogen), 1% penicillin-streptomycin (Invitrogen), 2 mM L-glutamine (Invitrogen), 0.1 mM non-essential amino acids (Invitrogen), 0.05 mM β-mercaptoethanol (β-ME, Sigma-Aldrich, St Louis, MO) and 10 ng/ml basic fibroblast growth factor (bFGF). All pluripotent stem cells were differentiated into CMs using our previously established protocol (Lin et al., 2012). All iPSCs were used for differentiation at passages from 30 to 40. Two clones of DMD-iPSC1 (23D1 and 23D2) were differentiated and data are representative for those two clones, respectively. Briefly, the following conditions were used for cardiovascular differentiation using the basal StemPro-34 (Invitrogen) medium: day 0-1, bone morphogenetic protein 4 (BMP4) (5 ng/ml); day 1-4, BMP4 (10 ng/ml), bFGF (5 ng/ml) and activin A (1.5 ng/ml); day 4-8, XAV 939 (Sigma-Aldrich) (5 μM) and vascular endothelial growth factor A (VEGFA) (10 ng/ml) and day 8-22, XAV 939 (Sigma-Aldrich) (5 μM). Differentiation cultures were maintained in a 5% CO_2_ and 5% O_2_ environment. All cytokines were from R&D Systems.

### Whole-transcriptome sequencing

Ion Torrent sequencing platform and related kits are all from Life Technologies. The cDNA library was prepared using an Ion Total RNA-Seq kit. Emulsion PCR was performed using Ion OneTouch 200 kit V2 on an Ion OneTouch machine. The enriched Ion Sphere Particles were loaded into an Ion 316 chip and sequenced using an Ion PGM 200 Sequencing kit on an Ion PGM machine. Gene expressions were estimated as fragment per kilobase of exon per million reads (FPKM) by cufflinks. Fold change calculation and two-tailed Student's *t*-tests were performed using Microsoft Excel. Function and pathway enrichment were analyzed using Ingenuity Pathway Analysis.

### q-RT-PCR analysis

Quantitative real-time RT-PCR (q-RT-PCR) was performed on a 7900HT Fast Real-Time PCR System (Applied Biosystems) with Fast SYBR Green Master Mix (Applied Biosystems). The cyclophinin G (*CYPG*) gene was used as an internal control. Results were analyzed with Microsoft Excel, with gene expression for control S3 iPSC-CMs arbitrarily set as 1. Primer sequences are described in supplementary material Table S1.

### Immunofluorescence

iPSC-CMs were seeded on gelatin-coated coverslips, and incubated with the following primary antibodies at a 1:200 dilution ratio (except for DMD antibodies): anti-human Troponin T (CTNT) (Lab Vision), anti-human DMD NT, Rod or CT (Leica, 1:20 dilution), anti-human DIABLO and anti-human C-CASP3 antibodies (Cell Signaling). This was followed by incubation with Alexa-Fluor-488-conjugated (Invitrogen) or Cy3-conjugated (Jackson Lab) secondary antibodies. Images were recorded with a Leica DMRA microscope (Leica).

### Propidium iodide staining

Day 22 EBs from control and DMD iPSCs were dissociated into single cells using 1 mg/ml Collagenase B (Roche) at 37°C for 30 min and followed by 0.25% Trypsin (Cellgro) at 37°C for 5 min. Cells were fixed with 75% Ethanol at −20°C overnight and stained with 100 μl PI staining solution (50 μg/ml PI, 50 μg/ml RNase in PBS) for 30 min at 37°C in the dark. An ACCURI C6 Flow Cytometry (FACS) (BD) analyzer was used to detect DNA fragmentation.

### Transmission electron microscopy

EBs were fixed using 2.5% glutaraldehyde in 0.1 M PBS (pH 7.4) for 60 min, followed by three washes with PBS (15 min each) and post-fixed with 1% osmium tetroxide containing 1% potassium ferricyanide for 1 h. The fixed samples were washed in PBS, dehydrated in an ethanol series (30%, 50%, 70%, 90%, 100% for 15 min each time) and then in propylene oxide for 10 min. Next samples were infiltrated with epon by a propylene oxide and epon series starting with 1:1 for 3 h, then three times in 100% epon (1 h each time). The samples were then embedded in molds containing 100% epon and polymerized at 60°C overnight, followed by being sectioned at ∼80 nm thickness and stained with lead citrate for 8 min. Electron micrographs were taken using a JEOL JEM-1011 transmission electron microscope (JEOL, Germany).

### Mitochondria staining

iPSC-CMs were re-plated on coverslips. Mitochondria were stained using MitoTracker^®^ Red CMXRos dye (100 nM, Cell Signaling) at 37°C for 30 min, fixed with 4% PFA, followed by DIABLO immunostaining. To measure mitochondria membrane integrity, we stained iPSC-CMs with MitoProbe™ JC-1 (Invitrogen) by following the manufacturer's staining instructions. FACS analysis of CMs post JC-1 staining revealed two populations, with green fluorescence indicating damaged mitochondria and red indicating healthy mitochondria.

### Western blotting

Day 22 iPSC-EBs were collected and total proteins were extracted with Laemmli Sample Buffer (Bio-Rad). We used the Mitochondria Isolation Kit for Cultured Cells (Pierce Thermo) for fractionating mitochondria and cytosol from iPSC-CMs. Samples were next treated and analyzed using a routine western blotting protocol from Bio-Rad. Antibodies against the N-terminus, Rod domain and C-terminus domain of DMD were all from Leica. The anti-GAPDH antibody was from Millipore. Antibody against human cytochrome *c* was from Biolegend. Antibodies against human DIABLO, human CASP3, CASP8 and CASP9 and XIAP were all from Cell Signaling. After 2 h of incubation with horseradish peroxidase (HRP)-conjugated secondary antibodies (GE healthcare), the signal was detected using ECL reagents (Bio-Rad). The intensity of bands was quantified using ImageJ (NIH, Bethesda, MD).

### Electrophysiological recording

Ionic currents were recorded using the whole-cell patch clamp technique as previously described (Han et al., 2014) using Axopatch1D, Digidata 1322A, and pClamp 9 (Axon Instruments) for data amplification, acquisition and analysis. Currents were elicited by a protocol of depolarizing potentials of −130 mV to 50 mV in 10 mV increments from a holding potential of −80 mV. Current densities were measured as the peak current for each potential pulse. Currents were normalized to the cell capacitance and expressed in pA/pF.

### Ca^2+^ imaging

Dystrophic iPSC-CMs were cultured with or without P188 (1 mg/ml, Sigma-Aldrich) for 7 days. CMs were incubated with a Ca^2+^ indicator (Rhod-2 AM, 5 µg/ml, Molecular Probes) at 37°C for 10 min. Intracellular Ca^2+^ transients were optically recorded with a high spatiotemporal resolution CMOS camera (Scimedia, Ultima). Data were analyzed using custom-made software (IDL).

### Data analysis

Data are shown as mean±s.d. Statistical analysis was assessed by a two-tailed Student's *t*-test, with *P*<0.05 considered statistically significant.

## Supplementary Material

Supplementary Material
