# Supplementary material for: Modeling and study of the mechanism of dilated cardiomyopathy using induced pluripotent stem cells derived from individuals with Duchenne muscular dystrophy
Source: Dis Model Mech. 2015 May 1;8(5):457–66. doi: 10.1242/dmm.019505 (PMC4415895; doi:10.1242/dmm.019505)
Supplement: Supplementary Material [file supp_8_5_457__index.html]

Modeling and study of the mechanism of dilated cardiomyopathy using induced pluripotent stem cells derived from individuals with Duchenne muscular dystrophy — Supplementary Material 

# Modeling and study of the mechanism of dilated cardiomyopathy using induced pluripotent stem cells derived from individuals with Duchenne muscular dystrophy

## DMM019505 Supplementary Material

**Files in this Data Supplement:**

- **Supplementary Material**
