# Supplementary material for: Modeling and study of the mechanism of dilated cardiomyopathy using induced pluripotent stem cells derived from individuals with Duchenne muscular dystrophy
Source: Dis Model Mech. 2015 May 1;8(5):457–66. doi: 10.1242/dmm.019505 (PMC4415895; doi:10.1242/dmm.019505)
Supplement: Supplementary Material [file supp_8.5.457_DMM019505.pdf]

a Detection of transgenes in iPS cells

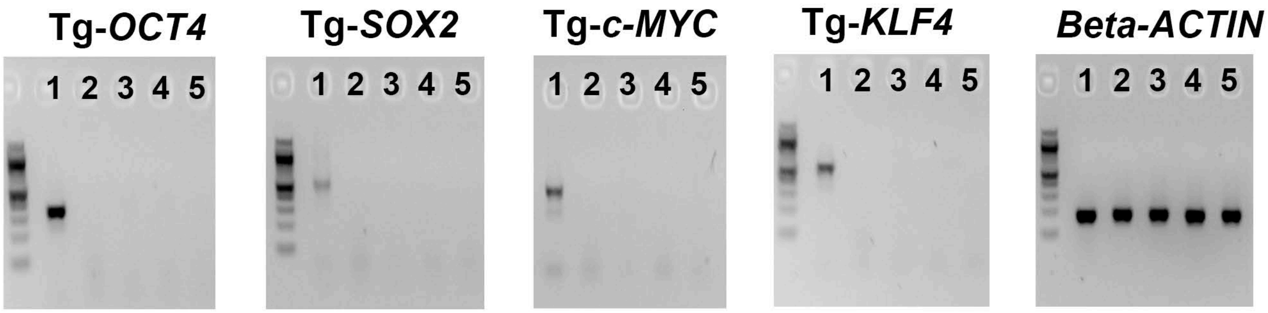

1. Human fibroblast cells transducted with retrovirus carrying each reprogramming factor
2. Control Y1 iPS cells
3. Control human S3 iPS4 cells
4. DMD-iPS1 cells
5. DMD15 iPS cells

b Detection of *DMD* mutations

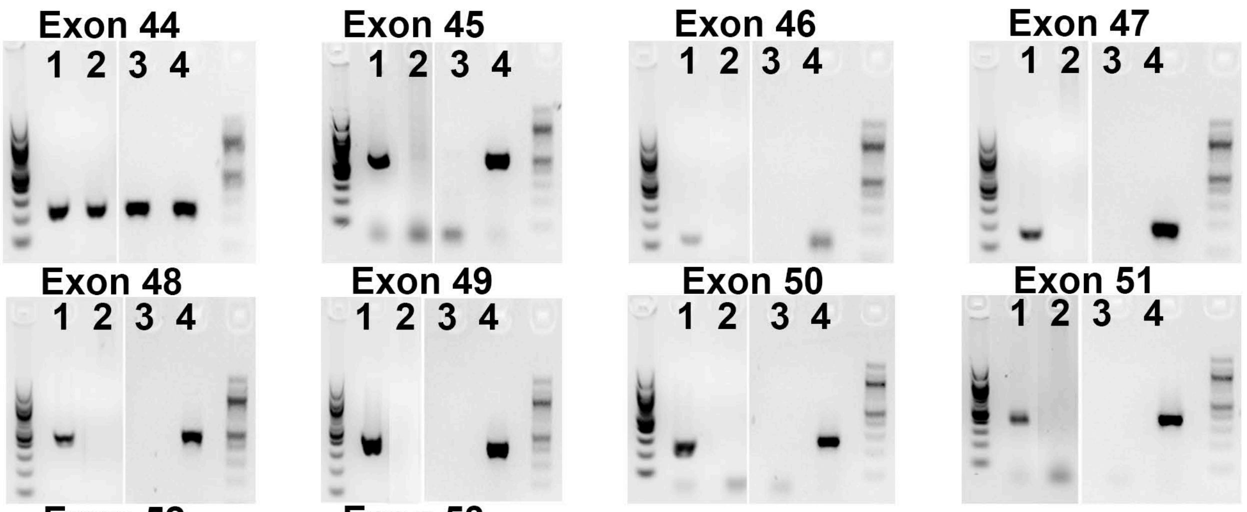

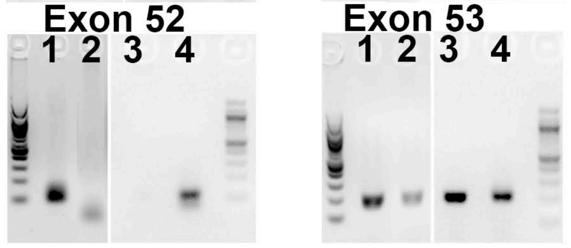

1. Control Y1 iPS cells
2. DMD-iPS1 cells
3. DMD15 iPS cells
4. Control human S3 iPS4 cells

Supplementary Fig. S1. Genotyping of iPSCs. (a) PCR detection of transgenic expressions of four reprogramming factors in the the established control and DMD iPS cells. BMD1 fibroblasts were transducted with retrovirus carrying OCT4, SOX2, KLF4 or c-MYC respectively. Total RNAs were collected 7 days post infection, followed with RT-PCR analysis. PCR primer sequence was shown in Supplementary Table S1. (b) PCR detection of exon 44 to 53 of the *DMD* gene. Exon 45-52 deletion was found in DMD-iPS1 and DMD15 iPSCs. PCR primer sequence was shown in Supplementary Table S1.

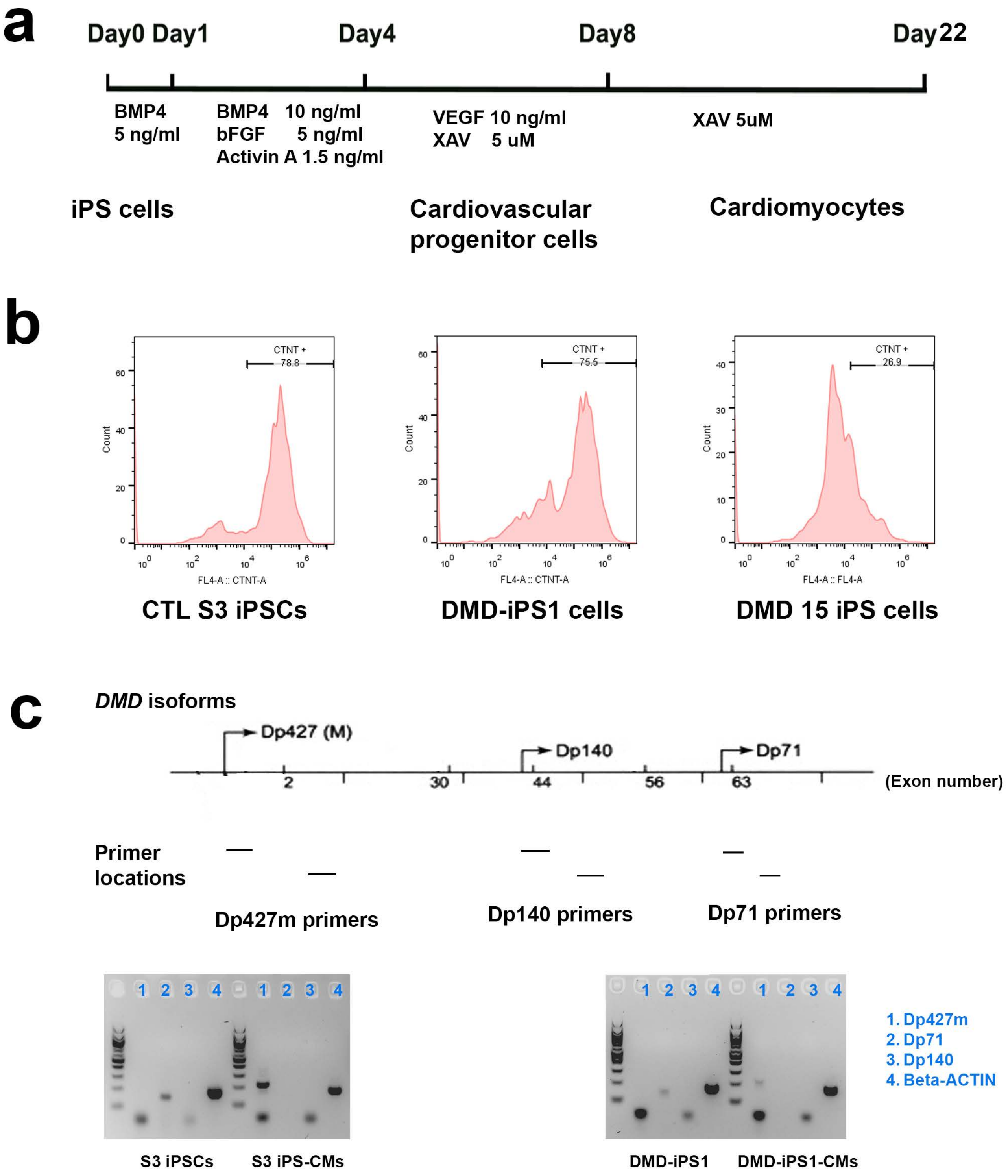

Supplementary Fig. S2. Cardiomyocyte differentiation from human iPS cells.
(a) Scheme showing our established high efficient cardiomyocyte diffentiation protocol from human iPS cells. (b) Ratios of CTNT+ cardiomyocytes from day 22 EBs of iPS cell differentiation. CTNT is a cardiomyocyte marker and the ratios of CTNT+ cells were quantified using FACS. (c) Detection of DMD isoform expressions in iPS cells and iPS-CMs. Three sets of primers were utilized to examine the expressions of Dp427m, Dp140 and Dp71 isoforms respectively.

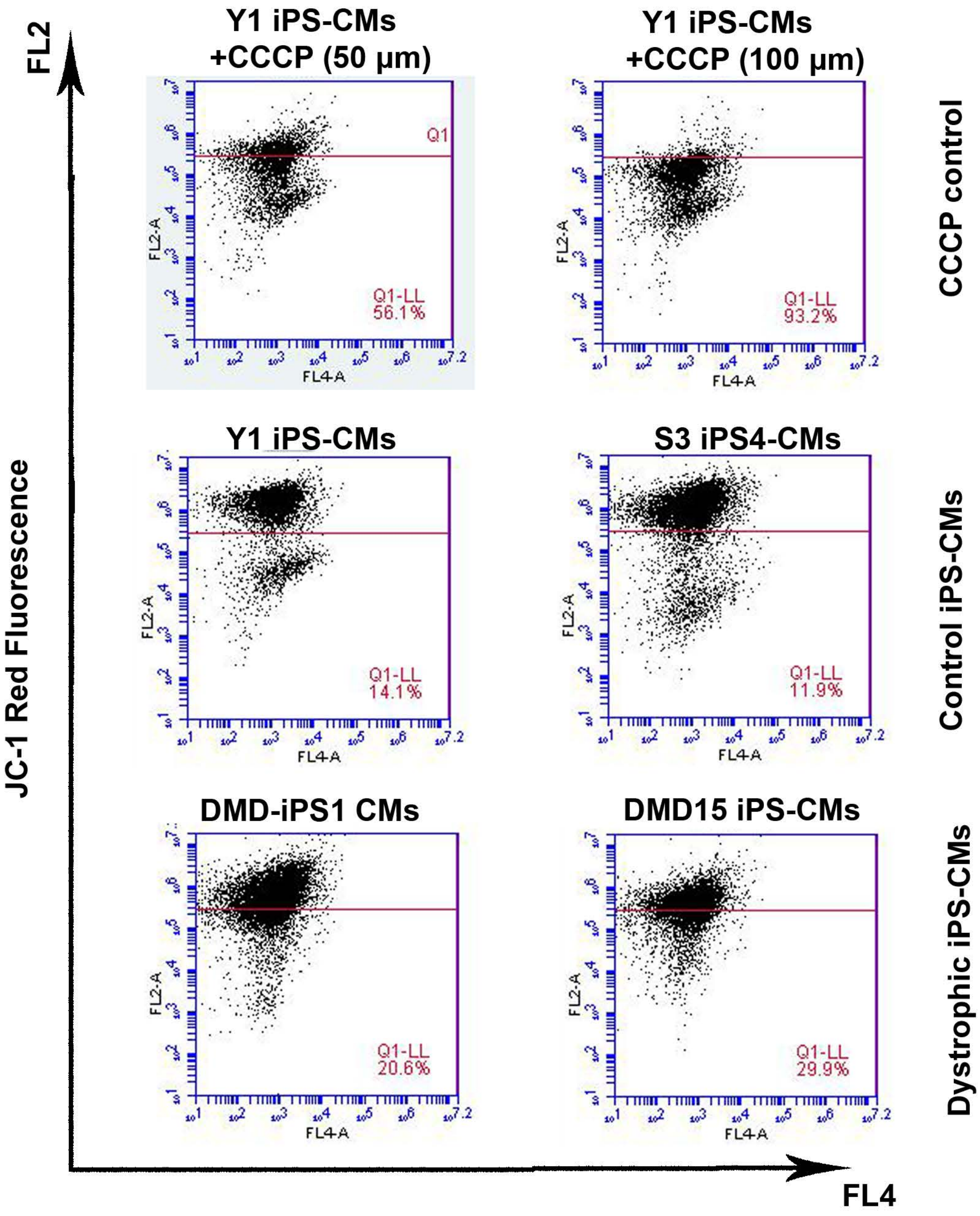

Supplementary Fig. S3. JC-1 staining for examining mitochondria health. Representative FACS results of JC-1 staining for control and DMD iPS cell-derived CMs. Red JC-1 indicates healthy mitochondria. And cells are negative for red JC-1 indicates damaged mitochondria. The healthy Y1 iPS-CMs were simultaneously incubated with JC-1 and a generic mitochondrial membrane depolarizer, CCCP (carbonyl cyanide m-chlorophenylhydrazone) (50, 100 µM final concentration), at 37°C, 5% CO2, for 15 min, followed with FACS analysis. CCCP control was used to confirm that the JC-1 response was sensitive to changes in membrane potential of iPS-CMs.

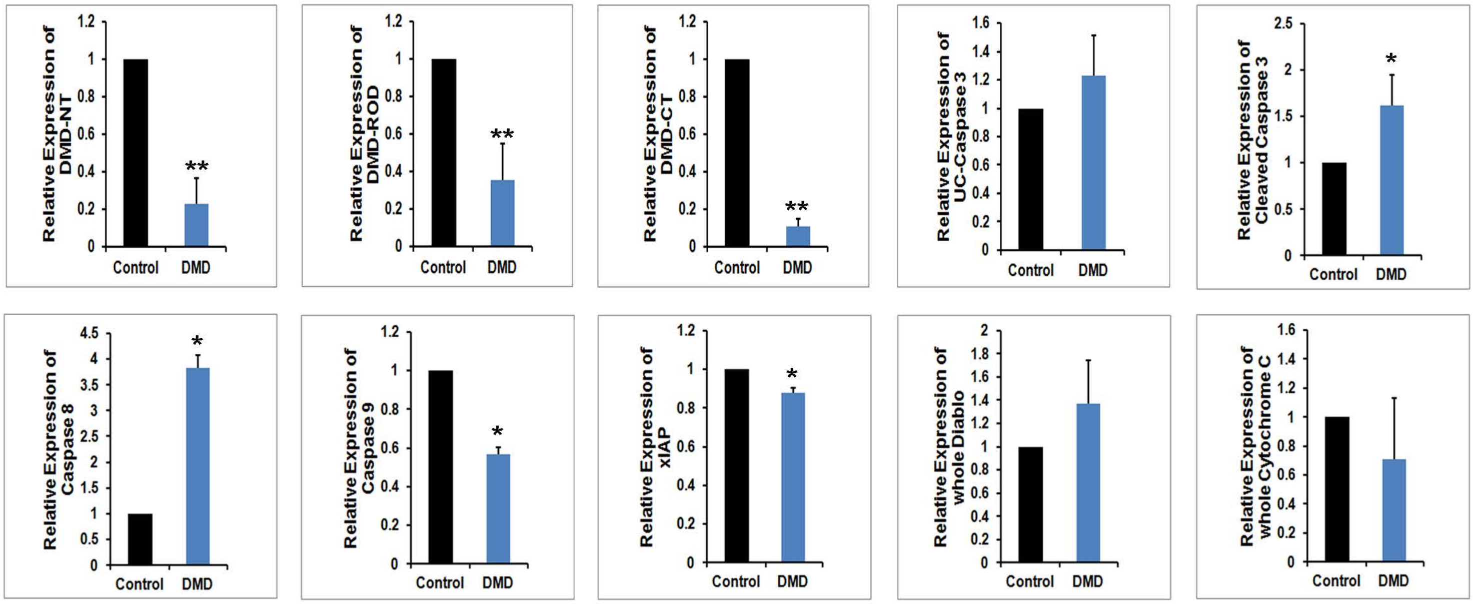

Supplementary Figure S4. Quantification of western blots. Band intensity was quantified by ImageJ and normalized to internal control GAPDH. Expressions of control CM were arbitrarily set as 1. Error bars represent SD of triplicate experments. *p<0.05. **p<0.01. (two tailed Student's t test)

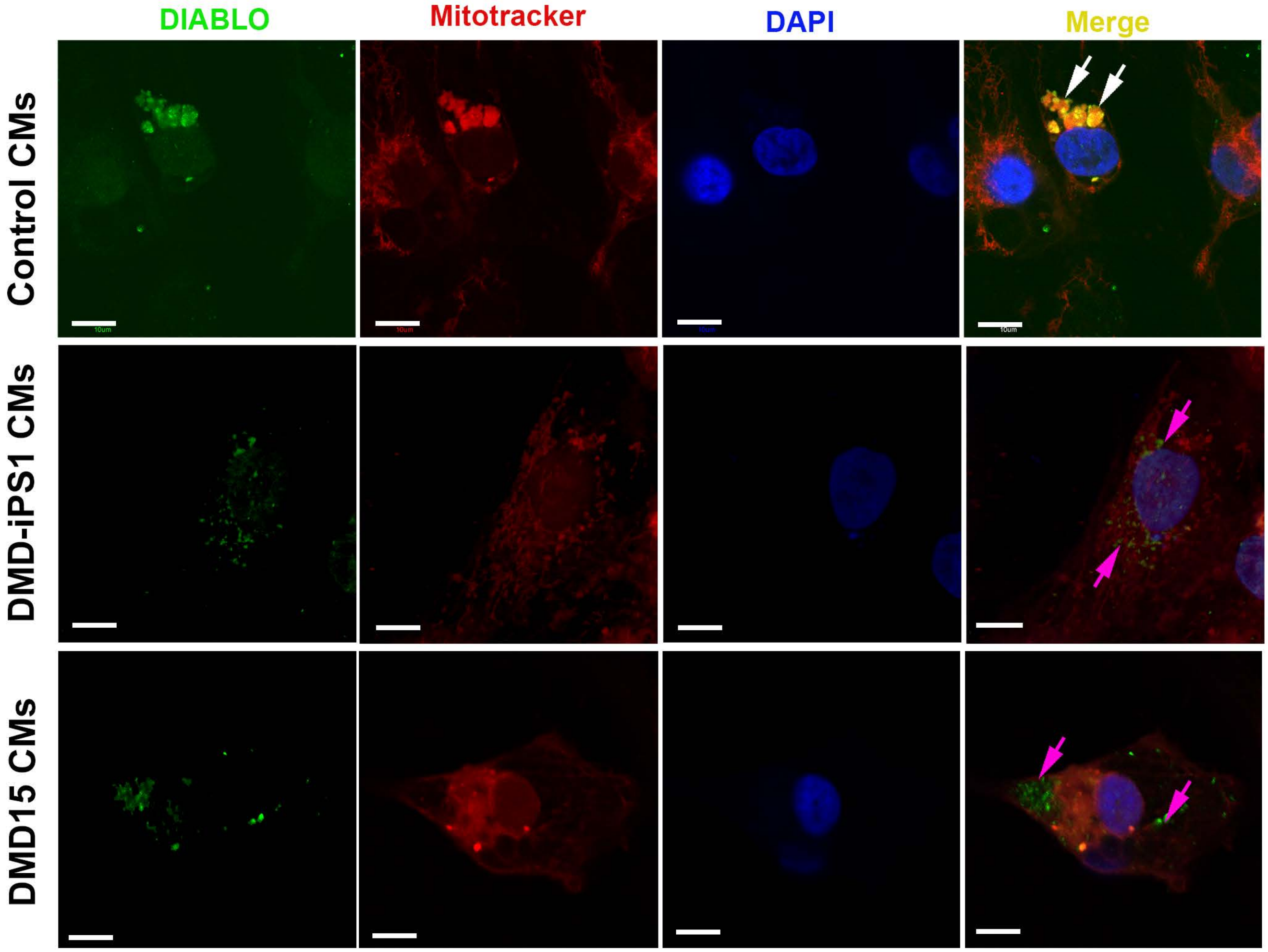

Supplementary Fig. S5. Co-staining of mitochondria and DIABLO. iPSC-CMs were stained with MitoTracker dye (red) to stain mitochondria and anti-DIABLO antibody (Green). White arrows indicated the DIABLO in mitochondria. Pink arrows indicated the cytosolic DIABLO released from mitochondria. Scale bars, 10 μm.

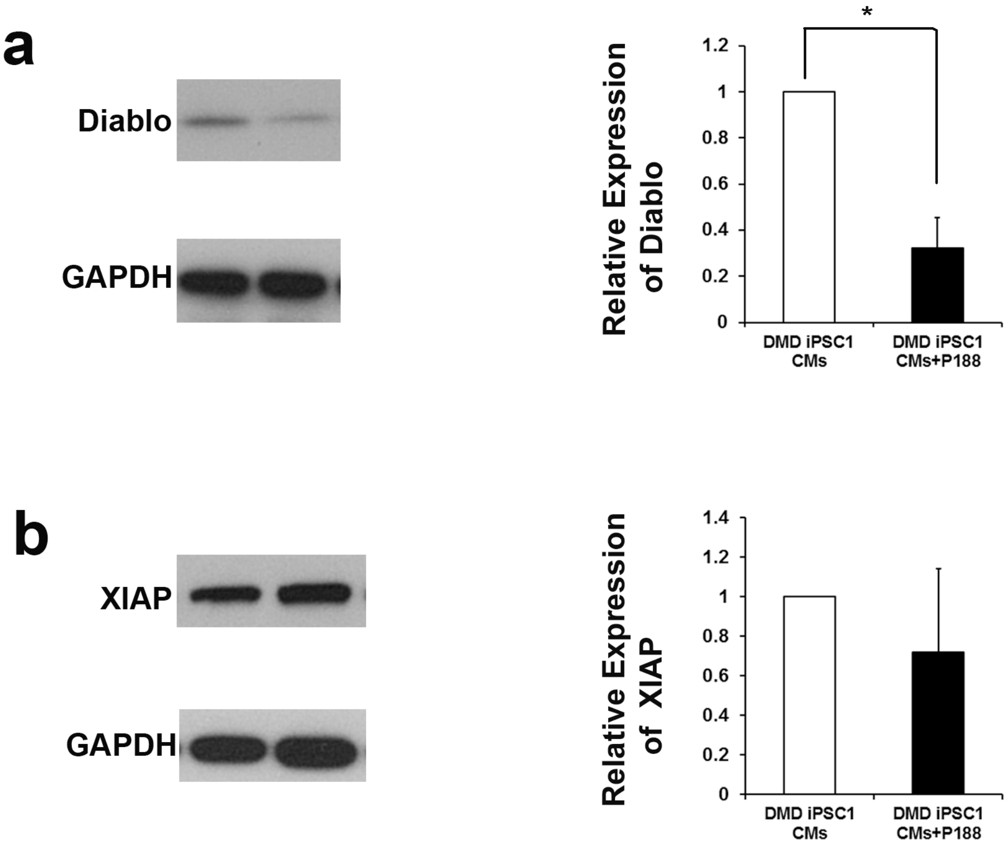

Supplementary Figure S6. Western blot analysis of DMD iPSC-CMs with vs. without P188 treatment (1 mg/ml) for 7 days. Representative images of western blot are shown in the left panel and statistical analysis of band intensity is shown in the right panel. Band intensity was quantified by Image J and normalized to internal control GAPDH. Expressions of untreated CM were arbitrarily set as 1. Error bars represent SD of triplicate experiments. *$p<0.05$, (n=3) (two tailed Student's t test) A. Diablo. B. XIAP.

Supplementary Table S1.
Primers used for Genotyping and QPCR and RT-PCR and Subcloning.

Primers For Genotyping		
**DMD EXON**	**FORWARD PRIMER**	**REVERSE PRIMER**
**EXON 44**	CTTGATCCATATGCTTTTACCTGCA	TCCATCACCCTTCAGAACCTGATCT
**EXON 45**	AAACATGGAACATCCTTGTGGGGAC	CATTCCTATTAGATCTGTCGCCCTAC
**EXON 46**	GCTAGAAGAACAAAAGAATATCTTGTC	CTTGACTTGCTCAAGCTTTTCTTTTAG
**EXON 47**	CGTTGTTGCATTTGTCTGTTTCAGTTAC	GTCTAACCTTTATCCACTGGAGATTTG
**EXON 48**	TTGAATACATTGGTTAAATCCCAACATG	CCTGAATAAAGTCTTCCTTACCACAC
**EXON 49**	GTGCCCTTATGTACCAGGCAGAAATTG	GCAATGACTCGTTAATAGCCTTAAGATC
**EXON 50**	CACCAAATGGATTAAGATGTTCATGAAT	TCTCTCTCACCCAGTCATCACTTCATAG
**EXON 51**	GAAATTGGCTCTTTAGCTTGTGTTTC	GGAGAGTAAAGTGATTGGTGGAAAATC
**EXON 52**	AATGCAGGATTTGGAACAGAGGCGTCC	TTCGATCCGTAATGATTGTTCTAGCCTC
**EXON 53**	TTGAAAGAATTCAGAATCAGTGGGATG	CTTGGTTTCTGTGATTTTCTTTTGGATTG
**Primers For QPCR**		
**ACTN1**	GAGAAGAGCATCGTCAACTAC	CAGGATCTGGTTCTCTACCT
**CASPASE 3**	GCCTGTAACTTGAGAGTAGATG	TGCCTCACCACCTTTAGA
**CASPASE 8**	TCAGAGGAGCAACCCTATT	CAGTCAGGATGGTGAGAATATC
**COL3A1**	GCATTCCTTCGACTTCTCTC	TTTCGTGCAACCATCCTC
**CYCLOPHININ G**	GAAGAGTGCGATCAAGAACCCATGAC	GTCTCTCCTCCTTCTCCTCCTATCTTTACTT
**DIABLO**	AGAGTACTTGAAGCTGGAAAC	TCTGACGGAGCTCTTCTATC
**MYL2**	GGTGCTGAAGGCTGATTAC	TTCTCCGTGGGTGATGAT
**MYL3**	CCTATGCTCCAGCACATTTC	CAACTTCTCCACTTCGTCTTC
**NPPA**	CTCTGTTTCCCCGCACGGTACCA	CTGCCCAGTGCCGCCTCTTTTT
**TPM1**	ACTGAAGTACAAAGCCATCAG	AGGGTCAGCTGGAGAATAG
**Primers For RT-PCR**		
**β-ACTIN**	CACCCAGCACAATGAAGAT	AGTCATAGTCCGCCTAGAAG
**DP427m**	TTGGTGGGAAGAAGTAGAGGACTG	GCAGTGCCTTGTTGACATTGTTCAG
**DP140**	ACACTAGCAATGGCAAAGCT	CTGCTCAGCTTCTTCCTTAG
**DP71**	CTCACTCCTCCACTCGTA	GACATTATTCAGGTCAGCTAAAG
**Tg-OCT4**	CCCTTTTTCTGGAGACTAAATAAA	CCCCAGGGCCCCATTTTGGTACC
**Tg-KLF4**	CCCTTTTTCTGGAGACTAAATAAA	ACGATCGTGGCCCCGGAAAAGGACC
**Tg-SOX2**	CCCTTTTTCTGGAGACTAAATAAA	GGCACCCCTGGCATGGCTCTTGGCTC
**Tg-MYC**	CCCTTTTTCTGGAGACTAAATAAA	CAACAACCGAAAATGCACCAGCCCCAG
**Primers For Subcloning**		
**XIAP**	ATCGGGATCCAAGGTGGACAAGTCCTAT	ATCGTCTAGACACATTCAATCAGGGTAATAAG

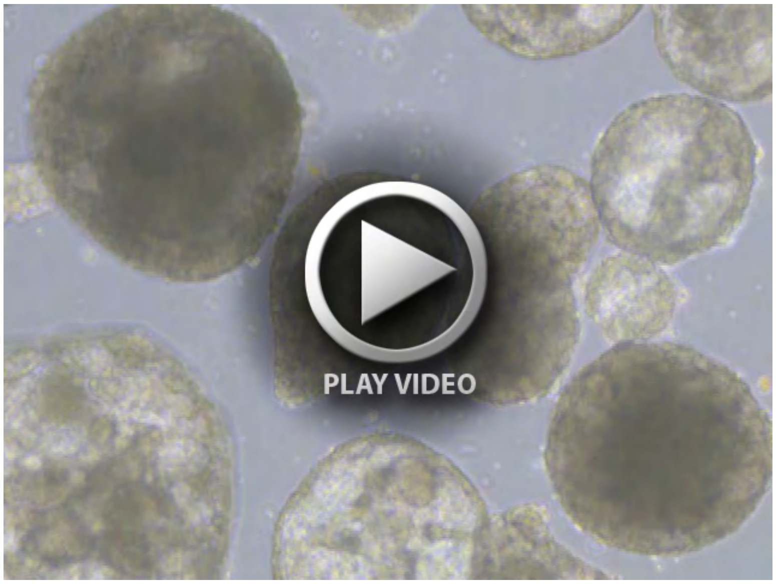

Supplementary Movie 1. Healthy control human Y1 iPS cell-derived beating day 22 EBs.

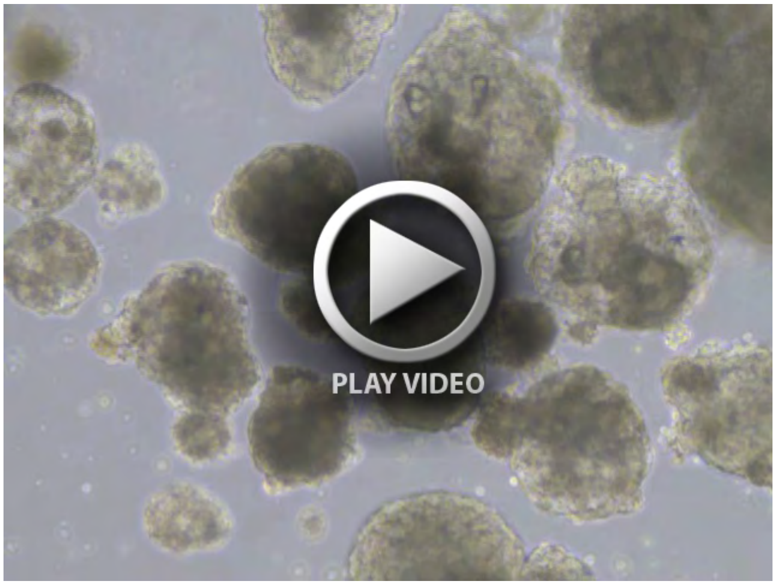

Supplementary Movie 2. DMD iPS1 cell-derived beating day 22 EBs.

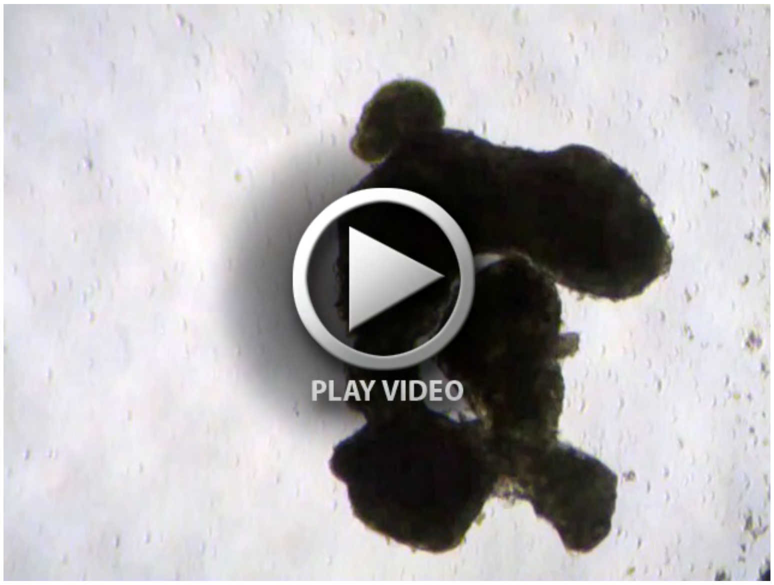

Supplementary Movie 3. DMD15 iPS cell-derived beating day 22 EBs.
